# Sodium Dodecylbenzene Sulfonate-Mediated Self-Assembly of Silk Particles from Formic Acid Solutions into Robust Films

**DOI:** 10.3390/polym17243277

**Published:** 2025-12-10

**Authors:** Rocco Malaspina, Martina Alunni Cardinali, Valeria Libera, Lucia Comez, Caterina Petrillo, Alessandro Paciaroni, Paola Sassi, Luca Valentini

**Affiliations:** 1 Department of Physics and Geology, University of Perugia, Via A. Pascoli, 06123 Perugia, Italyvaleria.libera@unipg.it (V.L.);; 2 Department of Chemistry, Biology and Biotechnology, University of Perugia, Via Elce di Sotto 8, 06123 Perugia, Italy; 3 CNR—Istituto Officina dei Materiali (IOM), Unità Perugia, Via Alessandro Pascoli, 06123 Perugia, Italy; comez@iom.cnr.it; 4 Civil and Environmental Engineering Department, University of Perugia, Strada di Pentima 8, 05100 Terni, Italy

**Keywords:** silk fibroin, self-assembly, FTIR analysis, hydrophobic interaction, phase separation, electrical properties

## Abstract

Silk proteins are versatile biopolymers well-suited to act as foundational components of a wide range of biomaterials. Rapidly gelling, self-assembling systems are especially valuable for drug delivery and biomedical applications. In this study, we present a way to induce the solid coaggregation of silk fibroin (SF) by adding the anionic surfactant sodium dodecylbenzene sulfonate (SDBS) into an SF solution prepared in formic acid (FA). SF films prepared by dissolving silk in CaCl_2_–FA and subsequently rinsing in water to remove CaCl_2_ were re-solubilized in FA with different content of SDBS. It was found that SF aggregation time is strongly modulated by the presence of SDBS. At increasing surfactant content, hydrophobic interactions between the SF chains and SDBS promote the formation of spherical coaggregates, whose size increases with surfactant concentration. FTIR analysis reveals that this process is accompanied by the formation of β-sheet structures, likely driven by hydrophobic interactions. This spontaneous liquid-to-solid phase transition promotes the formation of mechanically robust SF films with tunable electrical properties.

## 1. Introduction

Biopolymer-based particle systems have demonstrated utility across a wide range of applications, from biomedicine to smart materials for food [1,2,3,4,5,6,7,8,9,10]. Naturally derived polymeric particles spun from insect proteins such as silk represent a valid and sustainable alternative to synthetic materials [11,12,13,14,15]. Silk proteins can be extracted from silk fibers with different methods to obtain silk solutions that can be regenerated into biomaterials with different structural and morphological properties [14,15]. The conformation of liquid silk is indeed affected by various factors, such as pH, metal ions, temperature, and surfactants [16,17,18,19].

The above-mentioned parameters may play a crucial role in the phase transitions of silk fibroin (SF) where the precise mechanism governing its assembly into organized structures remains under debate. A key point is how the interaction between the surfactant and the protein architecture affects the phase transitions and the resulting assembly.

He et al., for example, highlighted the role played by Ca^2+^ ions in the fabrication of silk nanoparticles [20], mimicking the coagulation in silkworm glands. Regenerated silk particles derived from *Plodia interpunctella* silk through mechanisms of phase separation and nanoprecipitation were also reported [21]. Previously, in a pioneering study, it was reported that the acceleration of SF gelation was achieved by using an anionic surfactant, sodium dodecyl sulfate (SDS), as a gelling agent in a water environment [18]. However, SDS has poor solubility in acidic condition. This behavior can be ascribed to the low chemical stability of SDS in acidic conditions. In fact, the C-O-S bond is relatively weak, leading to the hydrolysis of the molecule with consequent formation of 1-dodecanol, which is poorly soluble in polar solvents because of its long aliphatic chain. Moreover, the hydrolysis process is accelerated at low pH because it is highly catalyzed by free protons.

In that study, it was proposed that the formation of solid coaggregates involves the linking of clusters formed by the accumulation of nanoparticles. More recently, it was reported that potassium ions influence silk nanoparticle formation, resulting in distinct physicochemical profiles [22]. Liquid–liquid phase separation (LLPS) also plays a critical role in the formation of SF particles. Ca^2+^ ions and anionic surfactant sodium dodecylbenzene sulfonate (SDBS) were found to trigger LLPS of silk solutions for the generation of robust protein-based fibers and adhesives [23].

However, these organized structures are usually obtained from the silk dissolution and regeneration process in a classical dissolving system, such as aqueous LiBr solution [1]. Although this method allows control over self-assembly conditions, it is a multistep and time-consuming process. Alternatively, our previous studies reported that silk can be directly dissolved in a CaCl_2_–formic acid (FA) solution, enabling the rapid production of silk materials in various ways [16,24,25].

Herein, we introduce a novel method to self-assemble SF particles into robust films with tunable bulk properties. This method involves the dissolution of SF films in FA and the sodium dodecylbenzene sulfonate (SDBS) surfactant. Hydrophobic interactions between SF and SDBS drive the formation of SF coacervates, whose characteristics can be modulated by the SDBS content. The spontaneous coaggregation–solid transition enhances particle cohesion, yielding mechanically robust bulk films. Key advantages of this methodology include the ease of operation, making it suitable for the efficient production of protein-based films with customizable functional properties.

## 2. Materials and Methods

### 2.1. Preparation of Silk Solutions

*Bombyx mori* silk fibroin was prepared according to a protocol as reported earlier [16,24,25]. Briefly, the silk cocoons were degummed with NaHCO_3_ and dispersed into 5 mL FA/CaCl_2_ solution (CaCl_2_ was at a weight ratio of 70/30 with respect to the silk amount (0.70 g)). SF films were produced by leaving the SF solutions to evaporate onto Petri dishes overnight, with subsequent annealing at 40 °C for 2 h. Then, SF films were directly immersed in water for 2 h to remove the CaCl_2_ and residual formic acid. The SF films were then air-dried for 24 h and redissolved in FA (5 mL) to obtain a calcium-free silk solution [26,27]. SDBS (purchased from Merck KGaA, Darmstadt, Germany) was dissolved in formic acid in six different concentrations (0.04–1.6 M) under gentle magnetic stirring. Then, equal volumes of SF (with and without CaCl_2_) and SDBS were mixed to obtain a final concentration from 0.02 to 0.8 M. The measured pH of the solutions varied from 2.7 for the SF solution to 1.5 for the SF + SDBS 0.8 M solution. A total of 2 mL of the mixed solutions was gently piped onto a quartz substate. Commercial SF aqueous solution was also used for investigating the role of pH on the time taken to form solid coaggregates; a total of 6.97 g of SDBS powder was added to 50 mL of deionized water and stirred for 1 h to dissolve completely. This produced an SDBS aqueous solution with a concentration of 0.4 M and a pH value of 11. To investigate the nature of intermolecular interactions responsible for SF aggregation, NaCl (0.5 M), that disrupts electrostatic interactions, was added to the SF + SDBS 0.8 M solution.

To monitor the coaggregation process, turbidity changes occurring during the formation of solid coaggregates were measured at the absorbance mode (550 nm) at different time intervals. The turbidity variation was defined by the change in optical density (OD). The optical microcopy was also used to investigate the SF film morphology after the evaporation of the FA. SF solutions with different SDBS concentrations were prepared by diluting the SF solution with formic acid. Equal volumes of SF and SDBS solutions were mixed at room temperature to characterize the phase diagram of the SF-SDBS coaggregation. Mixed samples were dropped onto glass slides and observed under an optical microscope (HRX-01, Hirox Co., Ltd., Tokyo, Japan) to determine the occurrence of the liquid–solid phase separation.

### 2.2. Characterization of Silk Films

Structural analyses of silk fibroin (SF) in the presence of sodium dodecylbenzenesulfonate (SDBS) were carried out using micro-Fourier transform infrared (micro-FTIR) spectroscopy in transmission mode and in attenuated total reflectance (ATR-FTIR) modality. The transmission measurements were performed using a Bruker Tensor 27 spectrometer coupled to a Hyperion 3000 microscope (Bruker Optics GmbH & Co. KG, Ettlingen, Germany) equipped with a 15 Cassegrain objective and a 64 × 64 pixel focal plane array detector. A total of 10 μL of each solution (SDBS in FA, SF in FA, mixed SF + SDBS in FA at the various concentrations) were deposited onto CaF_2_ windows. From each window, three maps consisting of 4096 spectra were acquired. From the maps, 50 spectra per each solution were selected as the reference data for the analysis of the secondary structure of silk fibroin. Each spectrum was corrected for background using segmented baseline subtraction, followed by vector normalization. The average spectra for pure SF and pure SDBS were obtained and used as reference spectra for further analysis. Second derivatives of each spectrum were calculated, and the content of β-sheets and disordered structures were evaluated based on the negative peaks. The analysis of the second derivative transformation has some advantages since it prevents spectral artifacts that could arise from the baseline correction and the need to choose a proper normalization criterion. Furthermore, it better evidences the Amide I and II subcomponents, resulting in a very effective discrimination of secondary protein structures. In the second derivative profile, the maximum of an absorbance peak is transformed to a minimum; thus, the negative intensities of the peak can be directly associated with the species concentrations. Assignments of the spectral components were performed following the indication described by Belton et al. [28]. ATR FT-IR spectra were collected using a Bruker Optics Alpha spectrometer equipped with a Globar source, a RockSolid interferometer, a KBr beamsplitter, and an RT-DLATGS detector (Bruker Optics GmbH & Co. KG, Ettlingen, Germany). A drop of each SF + SDBS in FA solution was deposited onto the ATR diamond crystal and allowed to dry at room temperature. Spectra were recorded during FA evaporation, over the 380–5000 cm^−1^ range by averaging 32 scans.

Inverse capillary velocity was determined from the fusion events of two coacervates [29]. We recorded the videos of fusion events, and each fusion was then analyzed frame-by-frame to obtain the aspect ratio (AR) at each point in time. AR was determined by drawing an ellipse and measuring the long and short axes of the ellipse. ImageJ version 1.54p was used for the analysis. The AR was then plotted versus time, and characteristic relaxation time τ was determined by fitting the following exponential function to the data:

(1) $$AR=1+({AR}_{0}-1)e^{-\frac{t}{\tau }}$$

where *AR*_0_ is the initial aspect ratio.

Zeta potential measurements were performed to investigate the influence of FA, and zeta potential measurements were conducted on SF in FA, SDBS in FA and SF + SDBS 0.8 M in FA, respectively.

Conductivity measurements were performed on dried films by an impedance analyzer (Keithley 4200 SCS). Electrical resistance values were obtained from the slope of the current–voltage characteristic. Finally, the morphology of the cross-section of the SF film with 0.8 M SDBS was investigated by scanning electron microscopy.

## 3. Results

**Figure 1:**
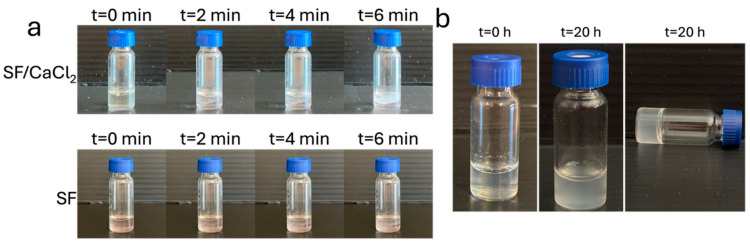
(**a**) Turbidity over time for SF solutions in FA prepared from SF/CaCl_2_ and SF films with SDBS dosed at 0.4 M. (**b**) Dynamic optical morphology of the aqueous solution of SF with 0.2 M SDBS and gel formation after 20 h.

As mentioned in previous studies, since the phase separation of SF is triggered by the content of Ca^2+^ [30], we investigate the role of Ca^2+^ in the SDBS/FA solutions. Before adding SDBS, the SF solution was transparent (Figure 1a). However, after SDBS addition, the solution showed a fast formation of aggregates, which were observed under an optical microscope (Figure 1a upper panel, Figures S1 and S2). On the contrary, when the SF formic acid solution without Ca^2+^ ions was mixed with SDBS, the SF/SDBS solution became homogeneously turbid (Figure 1a lower panel). This mimics the LLPS phenomenon of protein solutions in which protein molecules spontaneously separate into two different phases [18,31,32,33].

The role of pH in the phase separation was also investigated. SF aqueous solution mixed with SDBS at pH 11 and at room temperature exhibited a spontaneous sol–gel transition after 20 h (Figure 1b). This finding agrees with previous studies in which the formation of a solid gel within several hours is due to a micro-structure transition from random coil to β-sheet secondary structures [34]. Hereinafter, we investigated the SF formic acid solution without Ca^2+^ ions.

**Figure 2:**
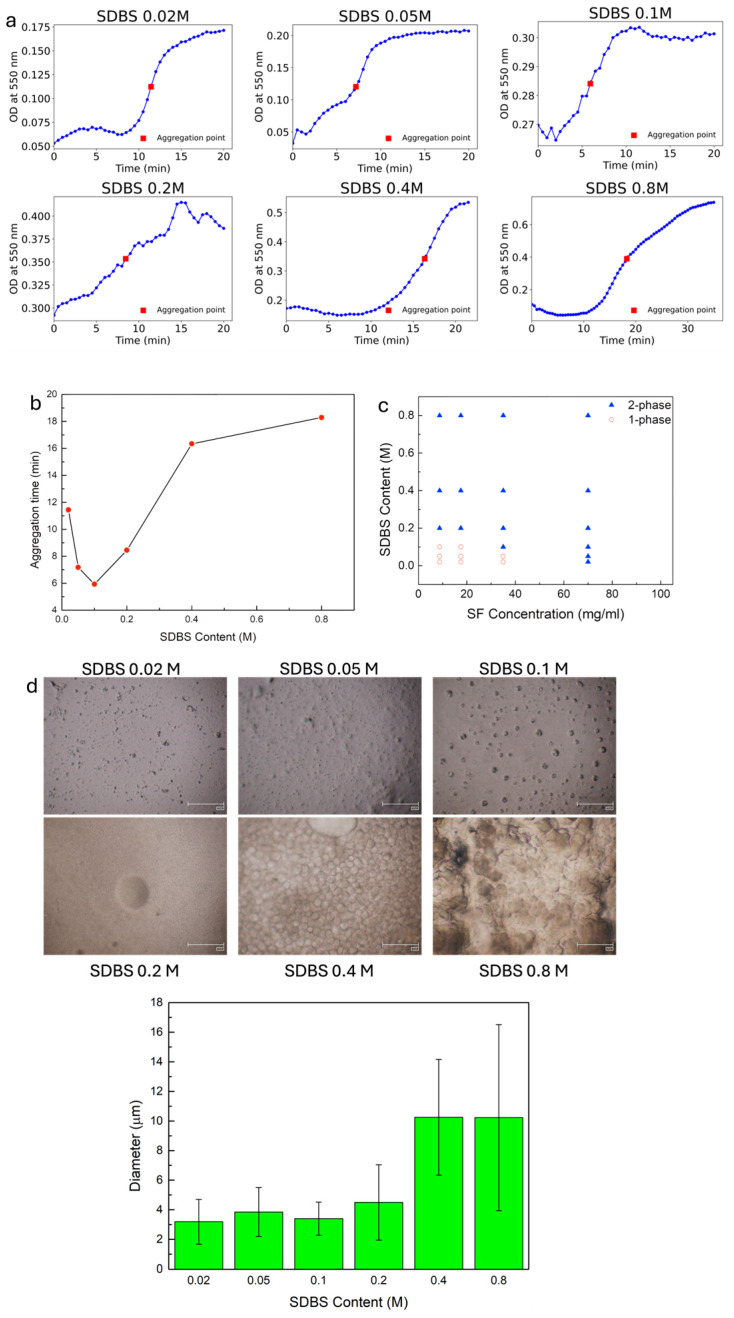
(**a**) Turbidity measurements for SF solutions in FA prepared from SF films with SDBS dosed at 0.02, 0.05, 0.1, 0.2, 0.4 and 0.8 M and (**b**) behavior of gelation time with SDBS concentration. (**c**) Phase diagram of silk solutions with SDBS. Filled triangles indicate solid–liquid phase formation; open circles indicate homogeneous solutions. (**d**) Optical microscopy of SF dried films prepared with different contents of SDBS and analysis of particle size (scale bars indicate 100 μm). Size calculations were performed using 150 particles derived from at least three different regions of interest.

The OD changes in the SF–SDBS mixtures with time are shown in Figure 2a. In each graph, the aggregation point was determined as the time point at which the optical density attains the mean between its minimum and maximum values. Initially, with increased SDBS lower than 0.1 M, the time for the appearance of solid coaggregates decreased. With SDBS increasing from 0.1 to 0.8 M, this time increased. These results indicate that the aggregation time is controlled by the SDBS content (Figure 2b). A similar behavior was observed by Wu et al. [18]. It was reported that there was a steep decrease in the gelation time of a water-based solution of silk fibroin upon addition of sodium dodecyl sulfate (SDS), followed by a slight increase in the gelation time after a certain concentration of SDS, in their case of 12 mM; this effect was due to electrostatic repulsion. In our case, we can assume that, even if electrostatic interactions play a minor role in the formation of SF-SDBS complexes, it is to be noted that still there are some negative charges in the SDBS solution, likely because a small part of the solfonic group is not protonated. Therefore, it is possible that at a higher SDBS concentration, the amount of negatively charged DBS- groups could be enough to cause an increase in the time of formation of SF-SDBS aggregates.

We expanded our analysis of the phase separation diagram to determine the boundary for a series of SF and SDBS concentrations (Figure 2c). The border between the clear solution (one phase) and turbid coacervate (two phases, e.g., solid–liquid) defines the critical concentrations that trigger the coaggregation [35,36]. At SDBS concentrations below 0.2 M, the mixture of SF and SDBS did not undergo phase separation at lower SF concentration, suggesting a critical SF concentration of 35 mg/mL for the SF/SDBS coaggregated system.

The turbidity observation can be ascribed to nucleation in hydrophobic blocks, which lead to the formation of SF/SDBS particles (Figure 2d).

Zeta potential measurements of solutions produced in FA (Table S1) indicate that the SDBS and SF + SDBS zeta potential ranged from −1.38 mV (SDBS in FA) to −0.76 mV (SF + SDBS in FA), indicating weak electrostatic interactions. To probe the role of hydrophobic interactions, NaCl was added to the SF + SDBS 0.8 M solution to disrupt electrostatic forces. The turbidity remained unchanged (Figure S3), indicating that hydrophobic interactions are the primary driving force behind phase separation. The impact of SDBS on silk particle size was then determined by optical microscopy. Higher contents in SDBS increased the particle size from 3 to 10 μm for lowest and highest surfactant content, respectively (Figure 2d, lower panel). Our findings are supported by a previous study by Kaplan and co-workers [18], who showed that the mechanism of aggregation is based on hydrophobic interactions between silk and the surfactant. Such interactions induce the unfolding, self-assembly, and association of fibroin chains. Amphiphilic SDBS molecules have a polar headgroup and a long aliphatic tail able to interact with the protein that is mainly hydrophobic. As a result, the associations not only among the SF chains but also between SF and the long alkyl chains of SDBS are favored by hydrophobic interactions. Agglomeration among molecules then occurs. The molecular aggregates in the solution can subsequently form numerous hydrophobic microdomains.

**Figure 3:**
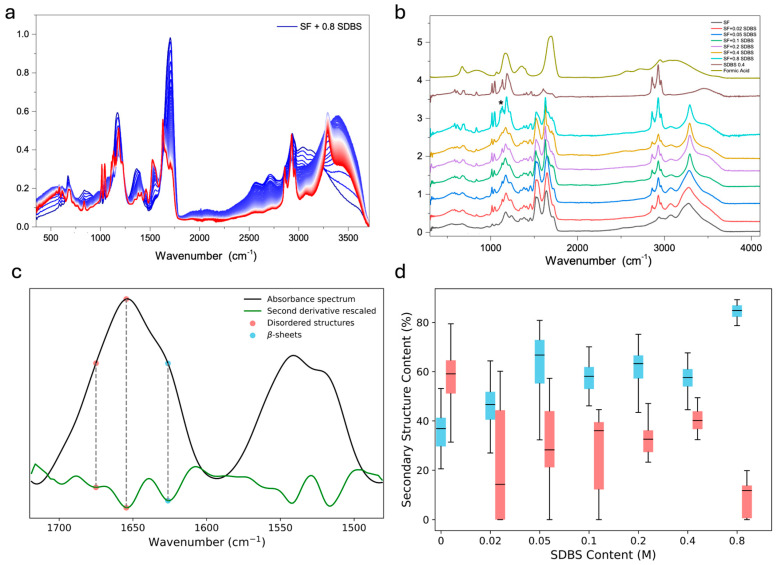
(**a**) FTIR spectra of the SF + 0.8 M SDBS sample collected during the desolvation process: the strong FA bands (blue spectra), especially the 1726 cm^−1^ peak, strongly diminish upon air exposure, accompanied by a pronounced increase in the intensity of the SF and SDBS signals (red spectra). (**b**) FTIR spectra of pristine SF film, SDBS, and SF film obtained from SDBS and FA solution. Asterisk denotes the 1090–1160 cm^−1^ region dominated by sulfonate/aromatic-coupled-sulfonate modes. (**c**) Second derivative transformation of the ATR-spectrum, evidencing peaks used to compare the secondary structures content between SDBS silk films and SF film alone. (**d**) Box plots showing the distribution of b-sheet (light blue) and random coil (light red) content as a function of SDBS concentration.

The dried films were then analyzed by infrared spectroscopy (Figure 3). Figure 3a reports the ATR-FTIR spectra of the SF + 0.8 SDBS sample collected during the film formation by drop-casting on the diamond crystal, clearly showing FA evaporation over time. Figure 3b shows the ATR-FTIR spectra obtained by drop-casting of the SF + SDBS in FA solutions at different concentrations after drying, along with the spectra of the pure FA and of SDBS dried from 0.4 M FA solution. FTIR spectra of SF shows three modes that are characteristic of the peptide bonds and more generally of the protein secondary and tertiary structure, namely Amide A, Amide II, and Amide I. Specifically, the amide A band, between 3310 and 3270 cm^−1^, is due to the stretching of the NH group [37]. The amide I band, mainly due to the stretching of the C=O group, is observed with a maximum at approximately 1650 cm^−1^. The Amide II band is related to the C-N-H bending and the CN stretching vibration and it is observed at 1528 cm^−1^ [37].

On the other hand, the FTIR spectrum of SDBS shows characteristic peaks of the C–H stretching vibrations at 2850, 2928, and 2956 cm^−1^, and a peak at ca. 1573 cm^−1^ assigned to the phenyl groups.

Evidence of potential formic acid residue is observed at 1726 cm^−1^, as evidenced by a comparison with the solvent spectrum (Figure 3b, olive green). Despite this minor contribution, the spectra of SF + SDBS films indicate that an increase in SDBS concentration results in a significant alteration in the SF amide bands. A pronounced enhancement in the signal intensity is observed at 1630 cm^−1^ and 1700 cm^−1^ (Amide I) and 3292 cm^−1^ (Amide A). These variations can be attributed to the rearrangement of the SF towards a more crystalline structure, dominated by the contribution of antiparallel β-sheets [38,39,40]. In addition, it is evident that at elevated SDBS concentrations (0.4 and more notably 0.8), discernible alterations manifest in the SDBS characteristic peaks, specifically within the 1090–1160 cm^−1^ region, which is usually dominated by sulfonate/aromatic-coupled-sulfonate modes (asterisk in Figure 3b). Indeed, the ATR spectra show that two additional narrow signals arise at 1122 and 1150 cm^−1^, alongside the two bands at 1134 and 1112 cm^−1^, likely assigned to CH aromatic in-plane bending combined with SO_3_^−^ asymmetric stretching [41,42]. This observation indicates that, at these concentrations, there is probably a splitting of the asymmetric SO_3_^−^ stretching envelope characteristic of aryl sulfonate groups, that could be attributed to the interaction between SDBS and fibroin, as the analogous concentration of dried SDBS in FA does not present the two additional signals.

The SF conformational changes induced by the addition of SDBS were further examined using micro-FTIR analysis. This approach enabled a more detailed assessment of microstructural heterogeneities within each sample (Figure 3c,d). The analysis of the β-sheet and random coil content (at 1628 and 1656 cm^−1^, respectively) revealed significant conformational variations in SF upon interaction with SDBS. The results are summarized in Figure 3c, where the box plots compare the content of β-sheets (blue) and random coils (light red) at increasing concentrations of SDBS.

In the absence of SDBS, the SF exhibited a lower degree of β-sheet structure than the SDBS silks. Indeed, all silk nanoparticles displayed an increase in total β-sheet content when exposed to SDBS, from about 50% in the less concentrated solution (0.02 SDBS) up to 80% in the more concentrated one (0.8 SDBS). According to previous works [22], our coacervates appear richer in β-sheets than the silk reference sample (SF prepared in FA and washed in water to remove CaCl_2_), consisting mainly of 30% β-sheet and 60% random coil. Particularly, micro-FTIR findings suggest that 0.04 M SDBS concentration may be sufficient to directly influence the structural rearrangement of silk. Formic acid plays a central role in mediating the coaggregation of SF and SDBS. By shifting the acid–base equilibria, FA promotes protonation of ionizable residues on the SF surface, thereby reducing the effective protein charge. On the other hand, the headgroup of SDBS is only weakly ionized and does not support the formation of classical micelles. Therefore, electrostatic stabilization becomes minimal, and hydrophobic interactions dominate. This environment favors direct association between SF and SDBS, leading to the formation of the spherical coaggregates observed in our system. During subsequent solvent removal, these densely packed regions reorganize into β-sheet-rich structures, consistent with the FTIR profiles. It is worth noting that combined use of macro-ATR spectroscopy and micro-FTIR mapping along with second derivative analysis provides a sensitive and reproducible approach for investigating the molecular mechanism underlying surfactant–protein interactions.

**Figure 4:**
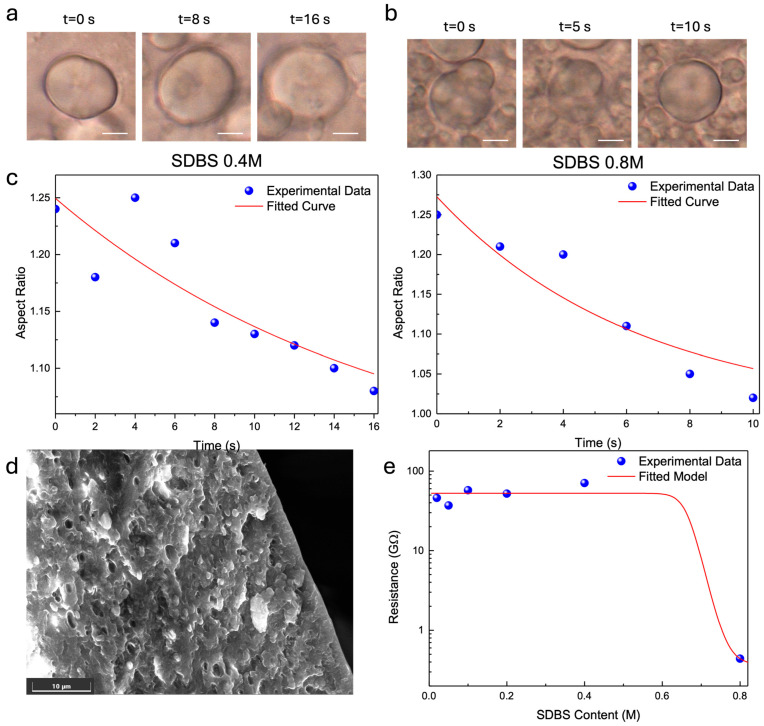
Fusion of two coacervates of silk with (**a**) 0.4 and (**b**) 0.8 M SDBS content. Scale bar is 5 μm. (**c**) Development of the aspect ratio over time from the fusion shown in (**a**,**b**) and data fit with the model reported in Equation (1). (**d**) Scanning electron microscopy image of the cross-section of the SF film with 0.8 M SDBS content. (**e**) Electrical resistance of SF/SDBS films as a function of the SDBS contents and data fit with semi-empirical model reported in Equation (2).

Since optical microscopy images suggest that the shape of the coaggregates is spherical, we studied the inverse capillary velocity (ICV) at different SDBS content. ICV was calculated from microscope videos taken from the fusion events of two coaggregates (Figure 4a,b). The images suggest that aggregates exhibit liquid-like viscous relaxation behavior where the surface tension drives them toward a spherical shape if they are transiently nonspherical.

For Newtonian liquid drops suspended in a lower viscosity medium, the characteristic time it takes two fusing droplets to assume a spherical shape, *τ*, is given by *τ* ≈ (*η*/*γ*)·*ℓ*, where *η* is the drop viscosity, *γ* is the surface tension, and *ℓ* is the characteristic length scale (size of drops) [43]. We plotted the aspect ratio vs. time of fusing SF drops (Figure 4c). This trend can be fitted by a simple exponential function, allowing us to determine the characteristic relaxation time *τ*.

The results showed a similar trend of silk solutions that had a faster relaxation time (≈6 s) for the highest SDBS content than that observed for 0.4 SDBS content (≈16 s). The high mobility observed and the ability to fuse by the droplets strongly indicate that silk forms liquid-like coacervates.

The evaporation rate of FA as a solvent also plays a role in microparticle dimension. During FA evaporation, droplets, transported by a flow, move from the inner region (see Movie S2). The droplets move in a radial direction, then the motion tends to stop as the droplet size becomes comparable to the surrounding drying droplets. As the evaporation proceeds, the surface tension difference across the solution–air interface changes due to the difference in concentration. Electron microscopy analysis (Figure 4d) reveals that the droplet in the inner region possesses a smaller diameter compared to the droplet near the edge. The difference in the diameters suggests a variation in the internal volume, which confirms differences in local variations in interfacial tension during droplet formation.

Understanding the electrical behavior of these silk-based systems requires disentangling the contributions of ionic conduction, interparticle contact, and molecular structure. A simplified semi-empirical model can be developed to describe the variation in electrical resistance (and, equivalently, conductivity) as a function of SDBS concentration for silk films containing particles differing in diameter and β-sheet content as follows:

(2) $$R\left(S\right)=R_{max}+\frac{R_{min}-R_{max}}{1+{\left(\frac{S_{0}}{S}\right)}^{p}}$$

where $R\left(S\right)\;$ is the resistance at SDBS concentration S(M), $R_{min}$ is the asymptotic low-resistance value at high S, $R_{max}$ is the plateau resistance at low S, $S_{0}$ is the transition concentration, and p  is the steepness exponent—large p  gives a very sharp transition. Fitting the model to the digitized points gave the following best-fit parameter values: $R_{min}=0.366\;G\Omega$, $R_{max}=52.56\;G\Omega$, $S_{0}=0.66\;M,$ and p=35.83.

In this model, the total electrical conductivity is expressed as the sum of two contributions: an ionic component, arising from dissolved SDBS and increasing sigmoidally with its concentration, and a network component, generated by physical contacts between silk particles. Network contribution strengthens as particle size decreases, due to a greater density of interparticle contacts at a fixed volume fraction, but is simultaneously weakened by the dispersing effect of SDBS on the particle network. At low SDBS concentrations, the resistance remains extremely high (in the GΩ range), reflecting both limited ionic conduction and a poorly connected particle network. Around a certain threshold concentration (approximately 0.66 M), a sharp increase in conductivity (i.e., a marked drop in resistance) is predicted. This transition is attributed to two concurrent effects of SDBS, namely (i) enhancement of the ionic conductivity of the medium, and (ii) improved interparticle contact, which promote additional charge transport pathways. It should be noted, however, that this represents a modeling assumption rather than a mechanistic interpretation.

## 4. Conclusions

This study presents a novel method to achieve coacervation in silk solutions using SDBS through hydrophobic interaction. The use of the SDBS-mediated coacervation strategy in formic acid offers advantages in terms of scalability of the process with respect to the use of aqueous silk solutions. We produced formic acid solutions from SF films and observed that SDBS surfactant induced phase separation. The presence of Ca^2+^ ions with SDBS promotes the fast formation of large silk aggregates, while SDBS in absence of calcium ions modulates the spontaneous formation of silk particles. Specifically, SDBS induced changes in coaggregation time, the formation of the secondary structures, tunable particle size, and particle net surface charge. The persistence of spherical silk particles in dried films indicates the stability of the combination of SF and SDBS due to strong hydrophobic interactions. The possibility of tuning the β-sheet content paves the way for the realization of silk hydrogels that benefit from the high stiffness of closely packed rigid microparticles. Thus, the combination of rigid microparticles and soft silk matrix in the hydrogel could result in a synergistic effect on high mechanical performance. Finally, this general approach imposes fewer limitations on the coaggregation process and raw material selection compared with conventional methods.

## Data Availability

The raw data supporting the conclusions of this article will be made available by the authors on request.

## Supplementary Materials

The following supporting information can be downloaded at: https://www.mdpi.com/article/10.3390/polym17243277/s1, Figure S1: Turbidity measurements for SF solutions in FA prepared from SF/CaCl2 films with SDBS dosed at 0.02, 0.05, 0.1, 0.2, 0.4 and 0.8 M; Figure S2: Optical microscopy of SF dried films prepared with different contents of SDBS from FA solutions of the SF/CaCl2 films; Figure S3: Picture of SF + SDBS 0.8 M solution before and after the addition of 500 mM NaCl; Video S1: Turbidity over time for SF solutions in FA prepared from SF films with SDBS dosed at 0.4 M; Video S2: Formation of SF particles in FA medium with a SDBS content of 0.4 M; Table S1: Values of the zeta potential measured for the SF, SDBS 1.6 M, and SF + SDBS 0.8 M solutions.
