# Supplementary material for: Sodium Dodecylbenzene Sulfonate-Mediated Self-Assembly of Silk Particles from Formic Acid Solutions into Robust Films
Source: Polymers (Basel). 2025 Dec 10;17(24):3277. doi: 10.3390/polym17243277 (PMC12737118; doi:10.3390/polym17243277)
Supplement: Supplementary file 1 [file polymers-17-03277-s001.zip › polymers-4031404-supplementary-done.pdf]

Supplementary information

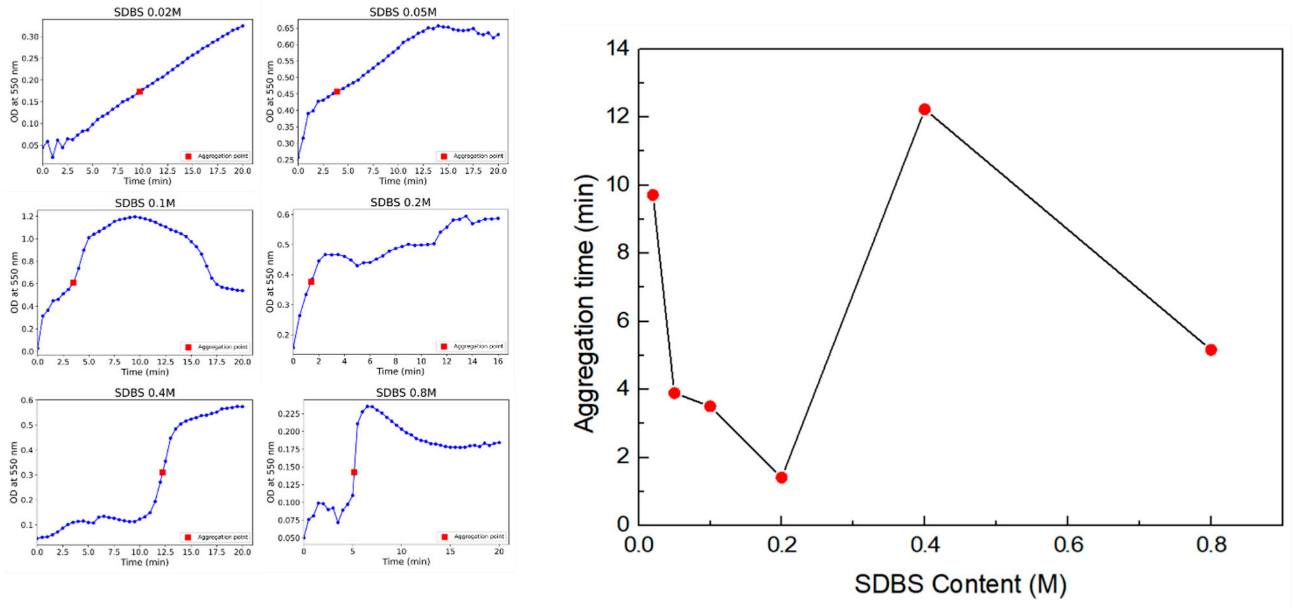

Figure S1. Turbidity measurements for SF solutions in FA prepared from SF/CaCl2 films with SDBS dosed at 0.02, 0.05, 0.1, 0.2, 0.4 and 0.8M.

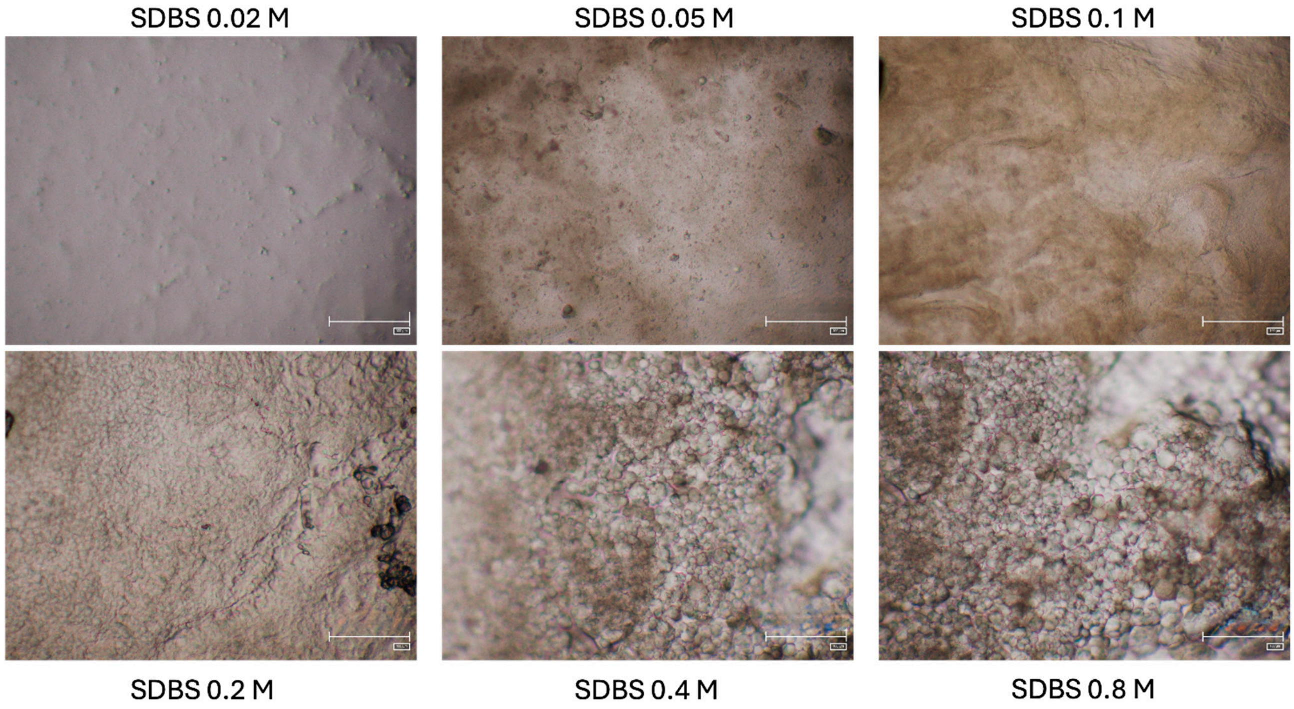

Figure S2. Optical microscopy of SF dried films prepared with different contents of SDBS from FA solutions of the SF/$CaCl_2$ films.

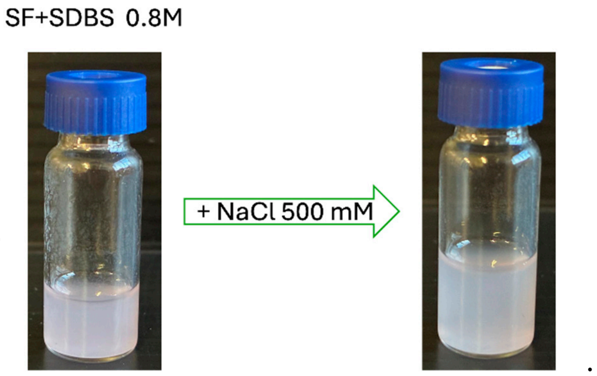

Figure S3. Picture of SF+SDBS 0.8M solution before and after the addition of 500 mM NaCl.

Video S1. Turbidity over time for SF solutions in FA prepared from SF films with SDBS dosed at 0.4M.

Video S2. Formation of SF particles in FA medium with a SDBS content of 0.4M.

Table S1. Values of the zeta potential measured for the SF, SDBS 1.6M, and SF+SDBS 0.8M solutions.

Sample	Zeta Potential (mV)
SF	0.69
SDBS 1.6M	−1.38
Mixed SF+SDBS 0.8M	−0.76
